# MSSA Thoracic Mycotic Aneurysm Repaired with TEVAR: A Case Report

**DOI:** 10.3390/reports8030184

**Published:** 2025-09-19

**Authors:** Umabalan Thirupathy, Vikramaditya Samala Venkata, Viraj Panchal

**Affiliations:** 1 Cheshire Medical Center, 580 Court Street, Keene, NH 03431, USA; sv.vikramaditya@gmail.com; 2 Department of Internal Medicine, Louisiana State University Health Science Center, Shreveport, LA 71103, USA

**Keywords:** MSSA, thoracic mycotic aneurysm, TEVAR

## Abstract

**Background and Clinical Significance:** Mycotic aortic aneurysm is a rare but life-threatening vascular condition characterized by infection-induced dilation or pseudoaneurysm formation in the aorta. The condition carries a high risk of rupture and mortality, especially in individuals with underlying cardiovascular disease, who have undergone recent vascular procedures, or with immunocompromising comorbidities such as diabetes. Its diagnosis is challenging due to its non-specific symptoms and often requires a high index of suspicion, especially in patients presenting with persistent fever and negative initial imaging. Early recognition and intervention are critical, as delayed treatment significantly worsens outcomes. **Case Presentation:** A 68-year-old male with a history of coronary artery disease, recent stent placement, and hypertension presented with two days of fever, chills, rigors, and a mild nonproductive cough. The laboratory findings were only significant for leukocytosis. The initial chest X-ray and non-contrast CT scans were unremarkable. He was admitted for presumed pneumonia and started on intravenous antibiotics. Persistent fever prompted further investigation with contrast-enhanced CT, which revealed a distal-aortic-arch pseudoaneurysm and mild mediastinal stranding. Blood cultures grew methicillin-sensitive Staphylococcus aureus (MSSA). Transthoracic echocardiogram was negative for endocarditis. The patient was transferred to a tertiary center, where repeat imaging confirmed a 1.5 cm pseudoaneurysm and a 4 mm penetrating atherosclerotic ulcer. After multidisciplinary assessment, he underwent thoracic endovascular aortic repair (TEVAR) and completed four weeks of intravenous cefazolin. Follow-up imaging showed successful aneurysm repair with no complications. **Conclusions:** Thoracic mycotic aneurysm is a rapidly fatal entity despite intervention. High clinical suspicion is necessary given its non-specific presentation. It is diagnosed most practically using CTA. In addition to antibiotics, TEVAR is gaining traction as a feasible and a safe alternative to open surgical repair (OSR).

## 1. Introduction and Clinical Significance

Mycotic aortic aneurysm is a rare but serious life-threatening condition caused by irreversible dilation of the aortic media resulting in the formation of an aneurysm or pseudoaneurysm secondary to an infection [1]. It is highly associated with a significant risk of rupture [2]. It commonly presents as a pulsatile, painful, and enlarging mass along with systemic features of infection [3]. Such infections are often insidious and can be challenging to diagnose, especially in patients who present with non-specific symptoms such as fever and rigors with initial negative imaging.

Despite the term “mycotic”, the most frequently implicated pathogens are typically bacteria, with Staphylococcus aureus being the most common [4]. Individuals who have an underlying cardiovascular disease, or who have undergone recent intravenous procedures or stent placements, are particularly susceptible to developing mycotic aortic aneurysm [5]. The bacteria invade a damaged arterial wall, which leads to inflammation, destruction, and eventually the formation of a pseudoaneurysm. Additional comorbidities like diabetes and hypertension further compromise vascular integrity [2].

We present a case of a 68-year-old man with multiple comorbidities and recent coronary stenting who was admitted with presumed pneumonia and persistent fever. Blood cultures revealed methicillin-sensitive Staphylococcus aureus (MSSA), and subsequent imaging revealed a distal-aortic-arch pseudoaneurysm.

## 2. Case Presentation

We describe the case of a 68-year-old male who initially presented to our emergency room (ER) with fever, chills, and rigors for 2 days. He also had a mild, nonproductive cough; otherwise, he denied shortness of breath. A thorough review of his systems was otherwise negative. Past medical history was significant for coronary artery disease requiring recent stent placement. He also had Class 3 obesity, hypertension, diabetes mellitus, diastolic heart failure, and chronic back pain.

He was febrile and hemodynamically stable on initial examination. His labs were concerning, indicating mild leukocytosis of 9.95 × 10^9^/L and elevated lactate at 3.6 mmol/L with corresponding elevated anion-gap metabolic acidosis; however, his renal and liver panel were within normal limits. He tested negative for respiratory viral panels. Initial chest X-ray and non-contrast computed tomography (CT) did not show evidence of pulmonary infiltrate. He was then admitted and started on an appropriate dose of intravenous antibiotics for pneumonia after obtaining blood cultures. Given his persistent fever, an extensive workup, including a contrast-enhanced CT (CECT) of the chest, abdomen, and pelvis, was planned.

**Figure 1:**
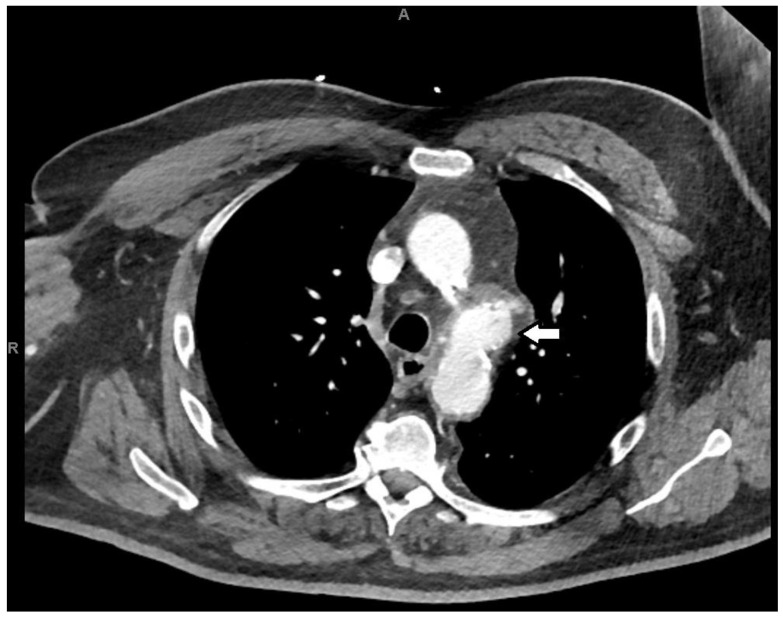
Cross-section of CT imaging with arrowhead highlighting the aneurysm of the distal aortic arch.

The imaging revealed a distal-aortic-arch pseudoaneurysm with mild adjacent mediastinal stranding, with indeterminate age and acuity (Figure 1). Blood cultures grew methicillin-sensitive Staphylococcus aureus in all four bottles. MSSA was cultured on blood agar culture media, and antimicrobial susceptibility was identified using an automated technique, the VITEK 2 method. A transthoracic echocardiogram failed to show evidence of infective endocarditis. Given concerns about a mycotic aneurysm in the setting of MSSA bacteremia, the patient was transferred to a tertiary center.

**Figure 2:**
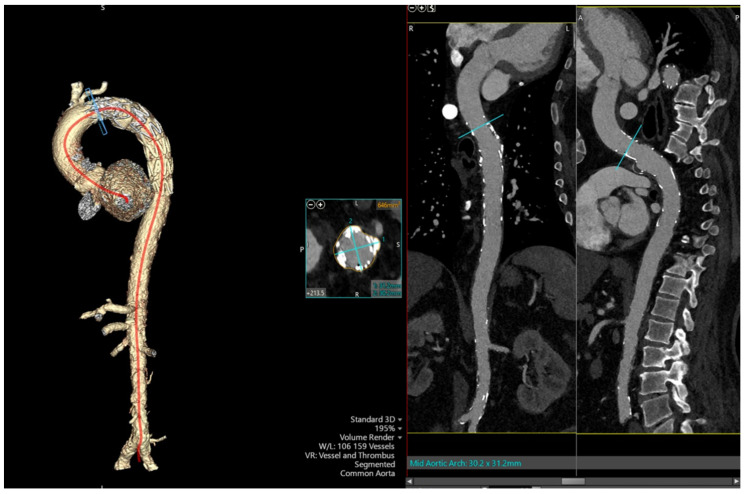
Cross-section of CT imaging with 3D reconstruction of the arch of the aorta and the descending aorta with a stent in situ (**left**), and coronal view of the stent graft from the arch of the aorta into the proximal descending aorta (**right**).

Repeated CECT Chest re-demonstrated the findings of distal-aortic-arch aneurysm with 1.5 cm irregular contrast-opacified outpouching/pseudoaneurysm abutting the left main pulmonary artery, in addition to a 4 mm penetrating atherosclerotic ulcer at the same level of the right aspect of the aorta. In addition, the thoracic aorta and its branch vessels were found to be atherosclerotic. After careful consideration of his medical comorbidities by a multidisciplinary team, thoracic endovascular aortic repair (TEVAR) was chosen instead of open surgery with debridement of the infected tissue. He was discharged with 4 weeks of intravenous Cefazolin. Remarkably, he remained clinically well, with a follow-up CECT Chest showing a repaired aneurysm and no evidence of endoleak on follow-up a month later (Figure 2). A repeat CECT Chest 6 months later showed that the aneurysm remained completely excluded and has not enlarged. Informed consent was obtained from the patient for the publication of this case, in accordance with ethical standards of the journal.

## 3. Discussion

The term “mycotic aneurysm” is a misnomer that Sir William Osler first attributed to a men dying from syphilitic aortic aneurysm in his Gulstonian lecture in 1885 [6]. One might argue that the term ”infectious aortitis” is a more pathologically appropriate nomenclature; however, “mycotic aneurysm” remains widely used. Thoracic mycotic aneurysms are rare and account for 0.2% to 1.4% of all aortic aneurysms [5]. Irrespective of the nomenclature and its rarity, it remains an invariably fatal entity [7].

The existence of a 4 mm penetrating atherosclerotic ulcer at the level of mycotic aneurysm in this patient, as evidenced in computed tomography with contrast, argues for an atherosclerotic plaque infection by MSSA as the likeliest etiology of the mycotic aneurysm. The development of atherosclerotic plaques or ulcers affects the integrity of the endothelial lining of the aortic intima, making it vulnerable to bacterial seeding in the setting of bacteremia [8]. Seeding from infective endocarditis was determined to be unlikely given the negative transthoracic echocardiogram for infective endocarditis.

Since the advent of antibiotics, infective endocarditis is no longer the most common etiology of mycotic aneurysm [9]. Other plausible mechanisms, albeit deemed less likely in our patient, include spread of an infection via the vasa vasorum supplying the aorta; a congenital abnormality, such as cystic necrosis or coarctation, again affecting the endothelial integrity of the aortic intima layer; and contiguous spread of infection from adjacent structures [5]. While the cause of initial bacteremia in our patient remains unknown, a review of the literature showed that this can occur in the setting of vertebral osteomyelitis, pneumonia, pancreatitis, or psoas muscle abscess [9]. In some cases of thoracic mycotic aneurysm, initial bacteremia with Staphylococcus aureus was even shown to predate the development of mycotic aneurysm by as much as 110 days [10].

The possible risk factors for myotic aneurysm in our patient are also risk factors for the development of penetrating atherosclerotic ulcers of the aorta, such as elderly age, hypertension, and diabetes mellitus [11]. Other risk factors for mycotic aneurysm are impaired immunity due to alcoholism, chronic glucocorticoid therapy, chemotherapy, cirrhosis, chronic hemodialysis, a post-transplant status, human immunodeficiency virus (HIV) infection, and malignancy [2].

Another clinical conjecture to be considered in our patient is that the recent coronary angiogram may have led to the development of the mycotic aneurysm at the distal arch of aorta. While coronary angiogram can theoretically cause bacteremia, only one previous case report associated coronary angiogram with a descending thoracic mycotic aneurysm [12,13].

The presence of respiratory symptoms in our patient confounded his clinical presentation and led him to be treated as a pneumonia patient initially. Thoracic mycotic aneurysm presents non-specifically but is most commonly characterized by fever (75%), thoracic and dorsal pain (60%), abdominal pain (20%), and chills (16%), as per a literature review [7]. In the largest retrospective thoracic mycotic aneurysm cohort study to date, involving 52 patients in Sweeden, 90% had fever. Thoracic mycotic aneurysm is also notable for causing Ortner syndrome by causing compression of the left recurrent laryngeal nerve in addition to dyspnea and dysphagia [14,15].

As in our patient, laboratory findings are significant for elevated inflammatory markers such as leukocytosis, elevated erythrocyte sedimentation rate (ESR), and C-reactive protein (CRP) [7]. Our patient also had a positive blood culture, which occurs in 50% to 85% of patients with mycotic aneurysm [2]. Common culprit organisms are Staphylococcus aureus, Pneumococcus, Escherichia coli, and Salmonella, in addition to Treponema pallidum, fungus such as Candida or Aspergillus, and mycobacterium tuberculosis [11]. The combination of staphylococcus and streptococcus alone accounts for 50–60% of mycotic aneurysms. Gram-negative organisms, especially Salmonella nontyphoidal strains, accounts for 30–40% of cases [5].

Despite the paucity of symptoms and non-specific laboratory investigations in our patient, the diagnosis was accelerated by the early utilization of imaging modalities. Computed tomography angiography (CTA) is the preferred imaging modality of choice in the diagnosis of myotic aneurysm [1]. CTA can be utilized rapidly in hemodynamic instability and to exclude aortic rupture. The most common diagnostic feature of mycotic aneurysm by CTA is periaortic stranding, as evidenced in our patient, which also occurs in 48% of cases. Diagnostic features include a contrast-enhanced saccular-shaped aneurysm wall with perianeurysmal fluid, or contiguous fluid, or, rarely, periaortic gas [5,16]. CTA can also rule out complications and be employed for serial monitoring of the mycotic aneurysm [17]. Late features include disrupted aortic calcification, indicating impending rupture, and contrast extravasation, indicating aortic rupture [18]. Compared to CT, magnetic resonance imaging (MRI) has a longer image acquisition time and slightly lower sensitivity and specificity due to its lower spatial resolution [17].

A transesophageal echocardiogram should be performed to rule out infective endocarditis and to diagnose aneurysm of the thoracic aorta [18,19]. 18F-fluorodeoxyglucose positron emission tomography (FDG-PET) can be used to assess the extent of inflammation and distant embolization and to monitor the response to treatment [2,18].

Given the rarity of thoracic mycotic aneurysm, there is no universal consensus available on its treatment. These patients should be immediately initiated on broad-spectrum empiric antibiotics. The use of antibiotics alone without surgical intervention carries a poor prognosis with mortality of almost 100% and should be reserved for patients who are moribund with palliative intent [2,5]. Experts opine that antibiotics should be continued for at least 6 weeks to 6 months with surveillance imaging [11].

Optimal treatment necessitates source control, which includes the option of open surgical repair (OSR) and thoracic endovascular aortic repair (TEVAR). TEVAR is ideally used as a bridge for open surgery. At present, it is impractical to design randomized trials to decide between these two techniques in treating a rapidly fatal entity such as thoracic mycotic aneurysm. Based on retrospective cohort studies, TEVAR is associated with improved short-term survival without late complications compared with OSR [20]. TEVAR can also be used as a bridge to OSR in unstable patients while being optimized. An observational study involving seven thoracic mycotic aneurysm patients who underwent definite intervention with TEVAR alone over the last 5 years concluded TEVAR to be safe and effective [8]. Despite earlier concerns about introducing graft material into an infected material, a paradigm change towards the feasibility and safety of TEVAR rationalized its use in our patient. More studies are needed to further elucidate which of these methods is most appropriate.

## 4. Conclusions

Thoracic mycotic aneurysm is a rapidly fatal entity despite intervention. High clinical suspicion is necessary given its non-specific presentation. It is diagnosed most practically using CTA. In addition to antibiotics, TEVAR is gaining traction as a feasible and a safe alternative to OSR.

## Data Availability

The original contributions presented in this study are included in the article. Further inquiries can be directed to the corresponding author.
